# Cloning of aquaporin-1 of the blue crab, *Callinectes sapidus*: its expression during the larval development in hyposalinity

**DOI:** 10.1186/2046-9063-8-21

**Published:** 2012-09-03

**Authors:** J Sook Chung, Leah Maurer, Meagan Bratcher, Joseph S Pitula, Matthew B Ogburn

**Affiliations:** 1 Department of Environmental Science, University of Maryland Baltimore County, Baltimore, MD, USA; 2 Department of Natural Sciences, University of Maryland Eastern Shore, Princess Anne, MD, USA; 3 Department of Natural Sciences, Savannah State University, Savannah, GA, USA; 4 Institute of Marine and Environmental Technology, University of Maryland Center for Environmental Science, 701 East Pratt Street, Columbus Center, Suite 236, Baltimore, MD, USA

**Keywords:** Aquaporin, Blue crab larvae, Ontogenetic variation, Osmoregulation, Salinity tolerance

## Abstract

**Background:**

Ontogenetic variation in salinity adaptation has been noted for the blue crab, *Callinectes sapidus*, which uses the export strategy for larval development: females migrate from the estuaries to the coast to spawn, larvae develop in the ocean, and postlarvae (megalopae) colonize estuarine areas. We hypothesized that *C. sapidus* larvae may be stenohaline and have limited osmoregulatory capacity which compromises their ability to survive in lower salinity waters. We tested this hypothesis using hatchery-raised larvae that were traceable to specific life stages. In addition, we aimed to understand the possible involvement of *AQP-1* in salinity adaptation during larval development and during exposure to hyposalinity.

**Results:**

A full-length cDNA sequence of aquaporin (GenBank JQ970426) was isolated from the hypodermis of the blue crab, *C. sapidus*, using PCR with degenerate primers and 5′ and 3′ RACE. The open reading frame of *CasAQP-1* consists of 238 amino acids containing six helical structures and two NPA motifs for the water pore. The expression pattern of *CasAQP-1* was ubiquitous in cDNAs from all tissues examined, although higher in the hepatopancreas, thoracic ganglia, abdominal muscle, and hypodermis and lower in the antennal gland, heart, hemocytes, ovary, eyestalk, brain, hindgut, Y-organs, and gill. *Callinectes* larvae differed in their capacity to molt in hyposalinity, as those at earlier stages from Zoea (Z) 1 to Z4 had lower molting rates than those from Z5 onwards, as compared to controls kept in 30 ppt water. No difference was found in the survival of larvae held at 15 and 30 ppt. *CasAQP-1* expression differed with ontogeny during larval development, with significantly higher expression at Z1-2, compared to other larval stages. The exposure to 15 ppt affected larval-stage dependent *CasAQP-1* expression which was significantly higher in Z2- 6 stages than the other larval stages.

**Conclusions:**

We report the ontogenetic variation in *CasAQP-1* expression during the larval development of *C. sapidus* and the induction of its expression at early larval stages in the exposure of hyposalinity. However, it remains to be determined if the increase in *CasAQP-1* expression at later larval stages may have a role in adaptation to hyposalinity.

## Background

Ontogenetic variation in salinity tolerance and osmoregulatory capacity may be directly related to patterns of dispersal and recruitment of animals in various aquatic habitats. In decapod crustaceans, adults of the blue crab, *Callinectes sapidus*, the green shore crab, *Carcinus maenas*[1], and the American lobster, *Homarus americanus*[2,3] are known to be strong hyper- and hypo-osmoregulators and inhabit a wide range of salinities. On the other hand, their embryonic and larval stages require high salinity water, possibly due to a limited osmoregulatory capacity [4]. Consequently, larvae are typically exported to higher salinity waters for larval development either by migration of females prior to spawning or rapid transport of larvae out of estuaries during ebb tides.

In estuaries such as the Chesapeake Bay, life stage-dependent osmoregulatory capacity and salinity tolerance may be the driving force underlying population structures of *C. sapidus*, resulting from migration of adult females to high salinity waters for spawning and the return migration of postlarvae (megalopae). First, adult females migrate to higher salinity areas near coastal waters after the pubertal- terminal molting and mating, where they spawn and release pelagic larvae [5]. These larvae largely spend seven-eight zoeal stages in coastal ocean waters [6]. However, upon molting to the megalopa stage, they migrate back to the coast and invade lower salinity estuarine areas where they metamorphose to the first crab stage [7]. Thus, the life cycle of *C. sapidus* presents a typical ontogenetic variation in salinity adaptation as osmoregulatory capacity and salinity tolerance are acquired during late larval development or the megalopal stage.

Salinity adaptation involves a complex process that entails dramatic changes in cell volume, ion transport, cellular metabolism, and whole-scale tissue remodeling. A large number of genes are involved in this osmoregulatory process in *Carcinus maenas*[8]. The aquaporin (AQP) family of water channels, small and very hydrophobic intrinsic membrane proteins, is critical in the physiological processes of water and solute transport for salinity adaptation [9]. AQPs are ubiquitous, being present in bacteria, plants, and animals. To date, 13 isoforms of the AQP family can be grouped into three subfamilies: aquaporins, aquaglyceroporins, and superaquaporins [10]. Among these three subfamilies, the aquaporin subfamily including AQPs 0, 1, 2, 4, 5, 6, and 8 is selective for water transport [11].

The involvement of aquaporins in salinity adaptation has been most studied for *AQP-1* in teleosts, although other aquaporins have been identified in this process. The expression of *AQP-1* is found in most organs of fish with high expression in gill, intestine, and kidney, where the expression levels change in response to different salinities. Acclimation to hyposalinity up-regulated *AQP 1a* expression in the gill of Atlantic salmon and black porgy [12,13]. On the other hand, acclimation to hypersalinity increased *AQP-1* expression in the intestines and kidneys of Atlantic salmon [13], and in the intestines of European eels [14], Japanese eels [15], and sea bass [16]. These studies demonstrate the potential for the involvement of aquaporins in the adaptation of *C. sapidus* to hyposalinity.

In view of the fact that adult females migrate to high salinity waters for spawning and that high salinity is required for larval development, we hypothesized that *C. sapidus* larvae may be stenohaline and have limited osmoregulatory capacity which compromises their ability to survive in lower salinity waters. We tested this hypothesis using hatchery-raised larvae that were traceable to specific life stages. In addition, we aimed to understand the possible involvement of *AQP-1* in salinity adaptation during larval development and during exposure to hyposalinity. We present evidence that larval stages Z2-6 exposed to artificial seawater (ASW) at 15 ppt showed significantly higher expression of the blue crab aquaporin orthologue *CasAQP-1* as compared to those exposed to 30 ppt. Molting percentage is much lower in ASW-exposed Z2-6 larvae as opposed to those reared at 15 ppt, suggesting that energy reserves are diverted to survival through osmoregulation under these conditions.

## Results

### Sequence analyses of *C. sapidus* aquaporin 1 (*CasAQP-1*)

**Figure 1:**
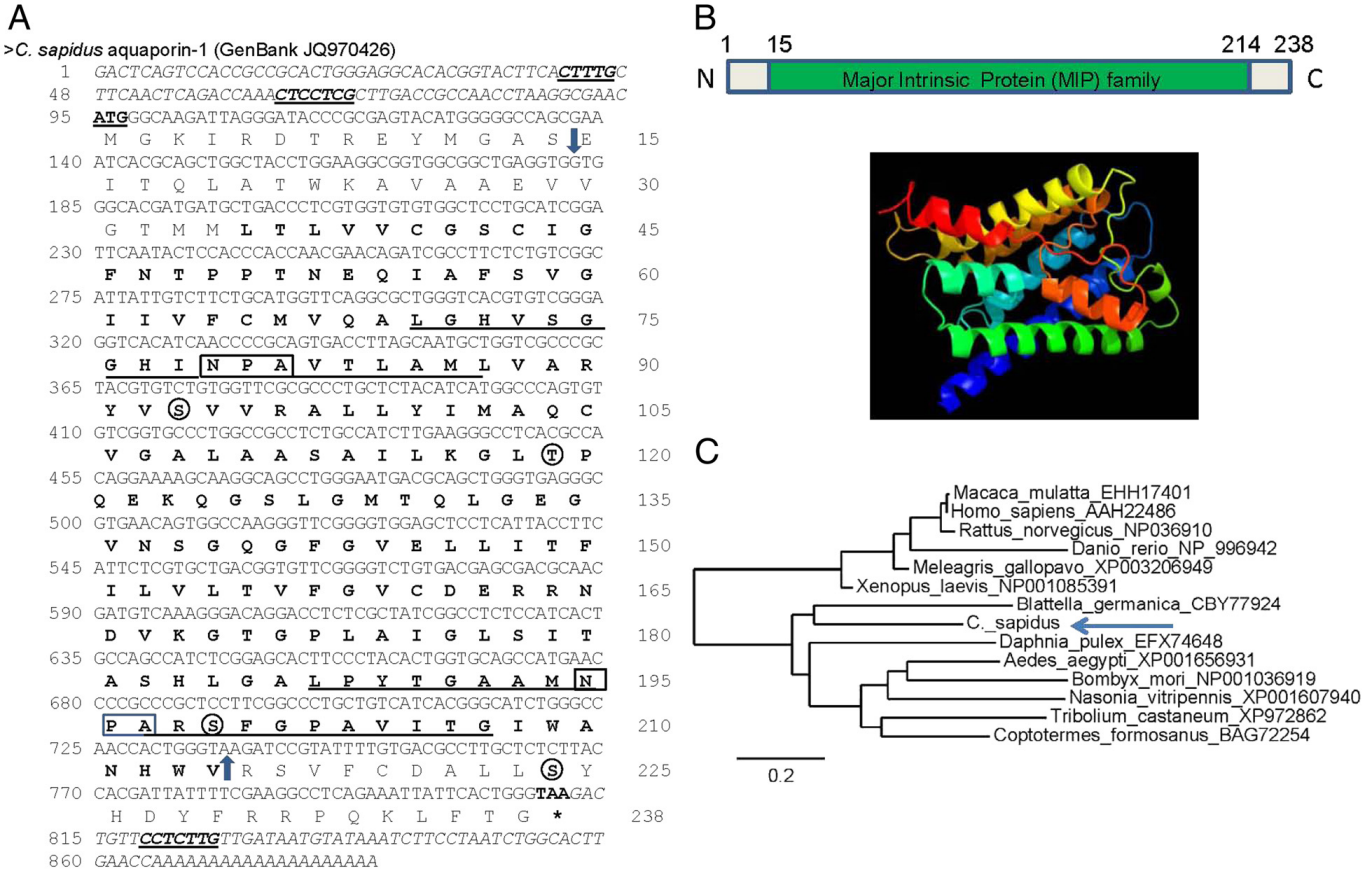
**A) The nucleotide and deduced amino acid sequences of cDNA encoding*CasAQP-1*(GenBank JQ970426) obtained from the hypodermis of*C. sapidus*.** The start codon (ATG) and stop codon (TAA) are in bold, and underlined and marked with a ‘*’, respectively. The first 94 nt and the last 63 nt are italicized for the 5′ and 3′ UTRs, respectively. Three putative regulatory sites, two in the 5′UTR (CTTTG and CTCCTCG) and one in the 3′ UTR (CCTCTTG), are shown in bold and underlined and the entire coding region was marked with arrows. The numbers shown at the right hand side are for nucleotides and the numbers at left hand side are for deduced animo acid. Putative phosphorylation sites: S and T are circled. B) Schematic diagram of a CasAQP putative aa sequence with the position of major intrinsic protein (www.ncbi.nlm.nih.gov/Structure/cdd). C) The phylogenetic analysis of AQP 1 was carried out (http://www.phylogeny.fr) and a phylogram was constructed using the neighbor-joining method with the deduced aa sequences of the following AQP-1: *C. sapidus* (**JQ970426**); *Coptotermes formosanus* (**BAG72254**); *Blattella germanica* (**CBY77924**); *Aedes aegypti* (**XP001656931**); *Daphnia pulex* (**EFX74648**); *Tribolium castaneum* (**XP972862**); *Nasonia vitripennis* (**XP001607940**); *Bombyx mori* (**NP001036919**); *Rattus norvegicus* (**NP036910**); *Xenopus laevis* (**NP001085391**); *Macaca mulatta* (**EHH17401**); *Meleagris gallopavo* (**XP003206949**); *Danio rerio* (**NP996942**); and *Homo sapiens* (**AAH22486**). The scale bar (=0.2) represents fixed mutations per amino acid position. D) 3D structure of CasAQP-1.

The nucleotide and deduced aa sequences of *C. sapidus* aquaporin-1 (CasAQP-1: GenBank JQ970426) are presented in Figure 1A. Both the 5^′^ and 3^′^ UTRs (italicized in Figure 1A) contained three terminal oligopyrimidine tracts (TOP) as a translation regulatory site: two located in the 5′ UTR and one located in the 3^′^ UTR (highlighted in bold and underlined in Figure 1A). The deduced amino acid sequence of CasAQP-1 does not contain the signal peptide (*P* = 0.068 by http://www.cbs.dtu.dk/services/SignalP). The Conserved Domain database (http://www.ncbi.nlm.nih.gov/Structure/cdd/) identified from the deduced amino a region from amino acid E_15_ to V_214_ of CasAQP-1 as a putative major intrinsic protein (MIP) superfamily member (Figure 1A, marked with arrows). The two highly conserved hydrophobic stretch regions, with two NPA boxes (boxed) that are involved in forming the water pore, are underlined. Four putative phosphorylation sites were predicted by NetPhos 2.0 Server (http://www.cbs.dtu.dk/services/NetPhos/) with a value >0.9 at three serine residues (S_93, 199_, _and 224_) and of 0.6 at one threonine residue (T_119_).

The 3D structure of CasAQP-1 (Figure 1B) was obtained using the 3D structure of c1ymgA (PDB) as the template showing that 91% of 217 deduced aa of CasAQP-1 were modeled with 100% confidence by the single highest scoring template. 10 α helices including six transmembrane helices, one β strand and 12 random coil secondary structures were predicted. Color rainbows indicates N- to C-termini of CasAQP-1.

A phylogram was generated with the deduced aa sequences of 15 different AQP-1 including seven vertebrates and 8 invertebrates (Figure 1C). The tree contains two separate clades: one for vertebrates and the other for invertebrates. CasAQP-1 was located close to AQP-1 of the cockroach, *Blattella germanica*, both of which were separate from the rest of the branch of insects and the water flea, *Daphnia pulex*.

### Spatial expression of *C. sapidus* aquaporin I (*CasAQP-1*)

**Figure 2:**
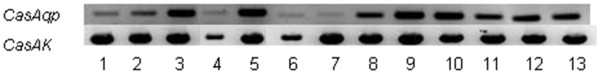
**Spatial expression patterns of*CasAQP-1*in various tissues except for ovary of a juvenile female*C. sapidus*.** Each tissue cDNA containing 12.5 ng of total RNA equivalent was amplified by an end-point PCR assay, while *AK* with a 350 bp amplicon served as a reference gene. 1 = eyestalk ganglia; 2 = brain; 3 = thoracic ganglia complex; 4 = hindgut; 5 = hepatopancreas; 6 = Y-organs; 7 = gill; 8 = antennal gland; 9 = abdominal muscle; 10 = hypodermis; 11 = heart; 12 = hemocytes; 13 = ovary.

The expression pattern of *CasAQP-1* in various tissue cDNAs prepared from a juvenile female *C. sapidus* at intermolt was examined with a pair of primers (CasAQP-1-3 F1 and -5R1, Table 1) amplifying the MIP domain (Figure 2). The ubiquitous *CasAQP-1* expression was found in all tissues tested, although there were some differences in expression levels. The thoracic ganglia complex, hepatopancreas, abdominal muscle, and hypodermis had the highest levels of expression, followed by progressively lower levels in the antennal gland, heart, hemocytes, ovary, eyestalk, brain, hindgut, Y-organs, and gill.

**Table 1:** **Primer sequences for cloning of the full-length cDNA of*C. sapidus*aquaporin-1 (*CasAQP-1*) and qRT-PCR assays**

	**Primer sequence (5′ to 3′)**
CasAQPdF1	GGNCAYATHWSKGGHGSHCA
CasAQPdF2	CAYATHAAYCCNGCNGTNAC
CasAQPdR1	GGNCCNAYCCARWANAYCCA
CasAQPdR2	AAMSWNCKRGCDGGRTTCAT
CasAQP-1-3F1	CTGGTCGCCCGCTACGTGT
CasAQP-1-3F2	CCCTGCTCTACATCATGGCCCAGTG
CasAQP-1-5R1	CCTGTCCCTTTGACATCGTT
CasAQP-1-5R2	CCGAACACCGTCAGCACGAGAATGA
CasAQP-1-QF	CTCACGCCACAGGAAAAGCAAG
CasAQP-1-QR	CAGCACGAGAATGAAGGTAATGAGG

‘d’ = degenerate primer; QF- and –R primers for qRT-PCR assay.

### Effects of salinity on molting and survival rate during the development of *C. sapidus* larvae

**Figure 3:**
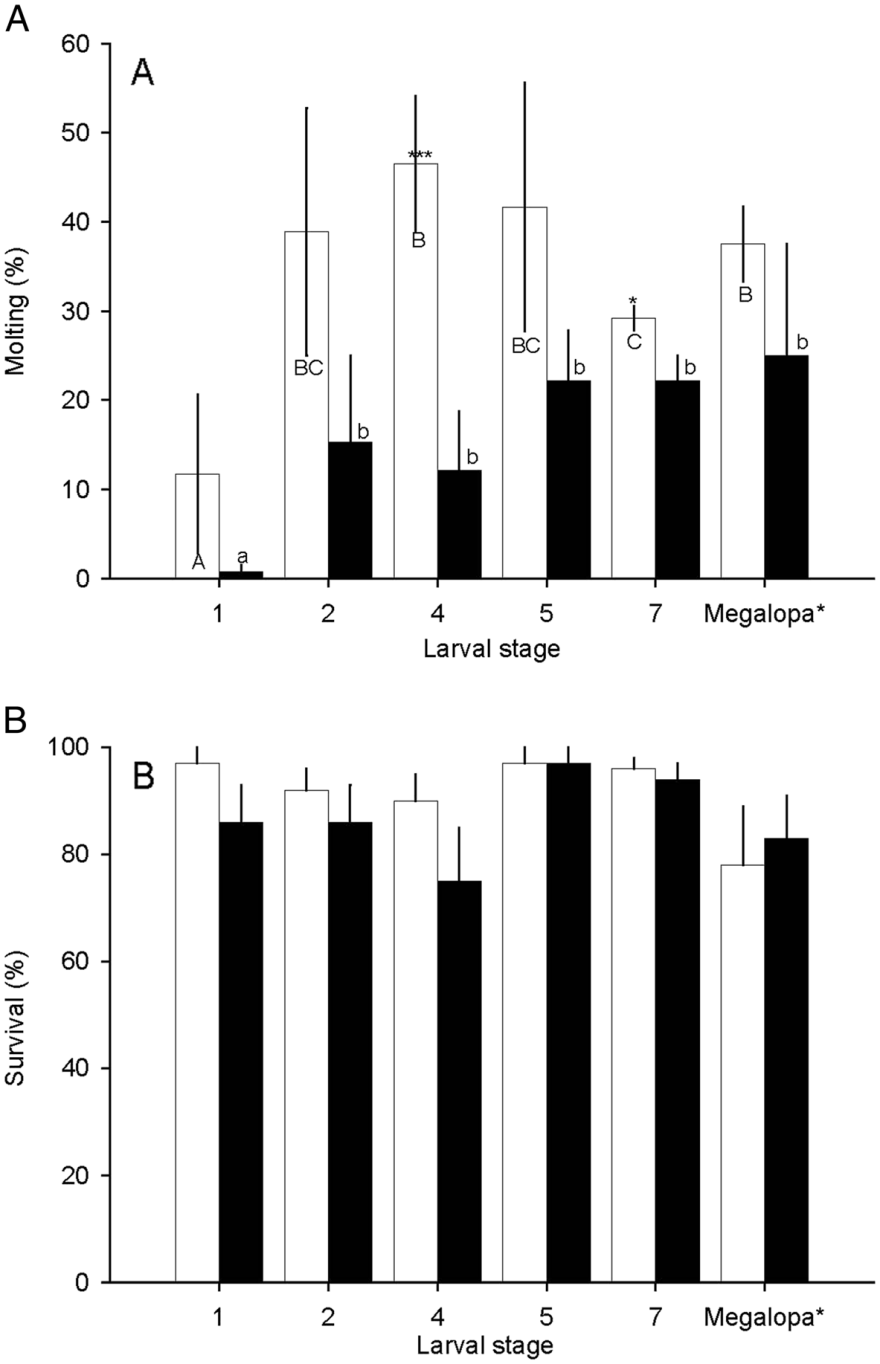
**The molting (A) and survival (B) rate of larvae exposed to artificial seawater at 30 (open bar) and 15 (closed bar) ppt after 96 hrs.** The data are presented as mean ± 1 SE (n = 5–9). Statistical significance among the larvae exposed to 30 ppt treated groups is determined at *P* <0.05 and noted in capital letters, and the 15 ppt treated groups are noted in lower case letters (one-way ANOVA and Tukey–Kramer multiple comparison tests). Statistical significance between the two groups was determined using the Student’s *t*-test and noted at *P* <0.05: *; *P* <0.001 <*** Unless specified, there was no statistical significance between the two groups or among the different larval stages.

The exposure of larvae to ASW at 15 ppt did not affect survival, but negatively affected the molting rate of *C. sapidus* larvae, as compared to controls held in ASW at 30 ppt (Figure 3A). In general, all larval stages (Z1-Z8) in ASW at 30 ppt showed molting rates varying from 12 ± 9% (Z1) to 47 ± 8% (Z4), in which Z1 had significantly lower molting rates than the rest of the larval stages. Larvae exposed to ASW at 15 ppt displayed a similar trend to those of the control but had much lower molting rates with 1 ± 1% at the Z1 stage, which increased significantly to a high rate of 13% (*P <*0.05) at Z2 onwards (Figure 3A). The statistical significance between the two groups is noted only at Z4 (*P* <0.001) and Z7 (*P* <0.05) larvae, due to a large variation at other larval stages.

Interestingly, however, there were no overall statistical differences in the survival of larvae among those exposed to ASW at 30 and 15 ppt (Figure 3B). At 30 ppt, the larvae at the first seven stages had 90- 97% survival, while Z8 to megalopae had survival of 78-97%. The larvae at Z1 to Z5 had 75-86% survival at 15 ppt, which was not significantly different from the controls with survival of 90-98%. The larvae at Z5-6 to Z8 also did not differ in survival at 15 ppt (83-97%) when compared to controls (78-97%).

### Effect of salinity on the expression of *CasAQP-1* and *arginine kinase (CasAK)* in *C. sapidus* larvae

#### *CasAQP-1* expression

**Figure 4:**
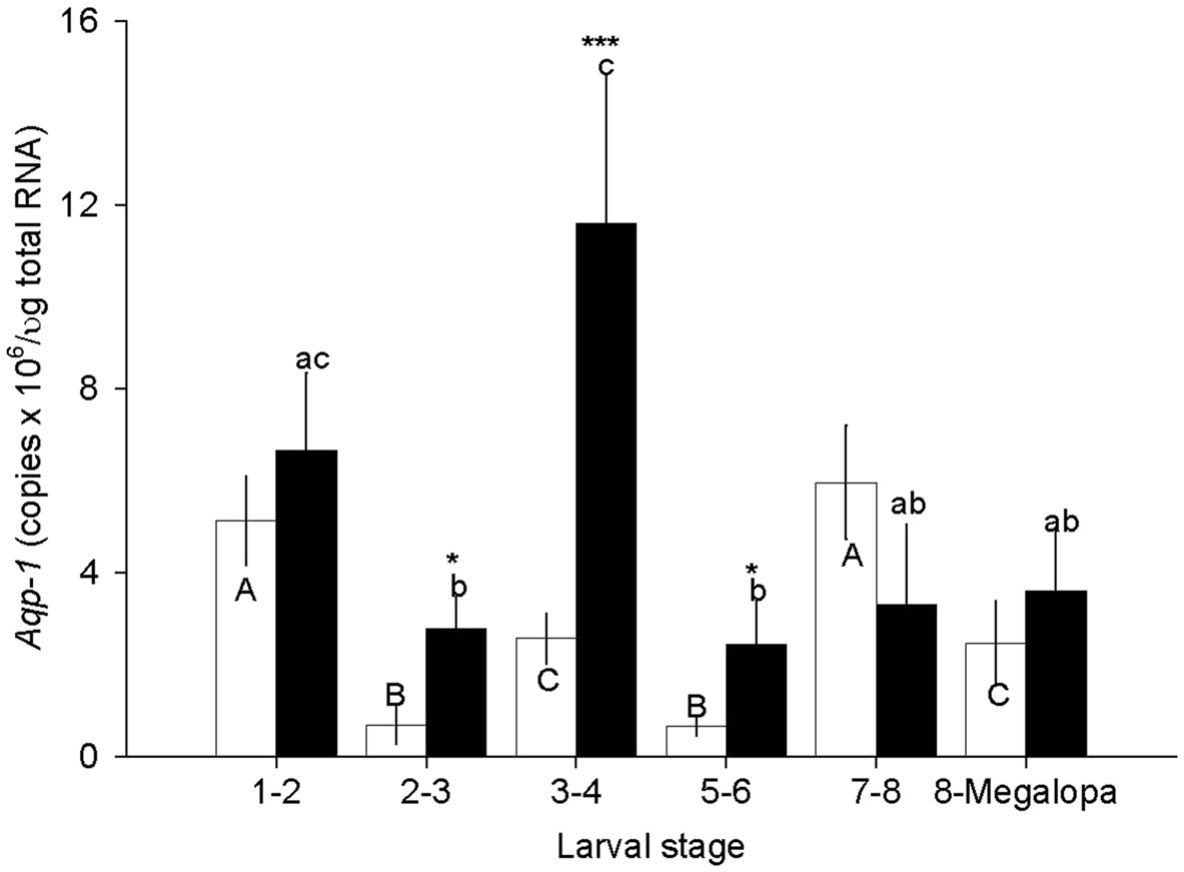
**Quantitative PCR (qRT-PCR) assays of*CasAQP-1*expressions in larvae at various developmental stages exposed to ASW at 30 (open bar) and 15 (closed bar) ppt.** The data are presented as mean ± 1 SE (n = 5–9). Statistical significance among the larvae exposed to 30 ppt treated groups is determined at *P* <0.05 and noted in capital letters, and the 15 ppt treated groups are noted in lower case letters (one-way ANOVA and Tukey–Kramer multiple comparison tests). Statistical significance between the two groups was determined using the Student’s *t*-test and noted at *P* <0.05: *; *P* <0.001 <***.

Experimental animals exposed to ASW at 30 ppt showed varying degrees of *CasAQP-1* expression from 0.7 ± 0.4 to 6.0 ± 0.9 x 10^6^ copies/μg total RNA (Figure 4). Larvae at stages Z1-2 and Z7-8 contained significantly greater expression of *CasAQP*-1: 5.1 ± 1.0 (n = 9) and 6.0 ± 0.9 x 10^6^ copies/μg total RNA (n = 6), compared to the larvae at stage Z2-3 (0.7 ± 0.4 x 10^6^ copies/μg total RNA, n = 7), Z3-4 (2.6 ± 0.5 x 10^6^ copies/μg total RNA, n = 5), and Z5-6 (0.6 ± 0.2 x 10^6^ copies/μg total RNA, n = 6).

Animals exposed to ASW at 15 ppt exhibited high levels of *CasAQP-1* expression ranging from 11.6 ± 3.2 to 2.4 ± 0.9 x 10^6^ copies/μg total RNA. Larvae at stage Z3-4 expressed the greatest amounts of *CasAQP-1* with 11.6 ± 3.2 x 10^6^ copies/μg total RNA (n = 5), which was followed by larvae at stage Z1-2 with 6.7 ± 1.7 x 10^6^ copies/μg total RNA (n = 7). At stages Z2-3 and Z5-6, the larvae show markedly less expression of *CasAQP-1* with 2.7 ± 0.7 x 10^6^ (n = 7) and 2.4 ± 0.9 x 10^6^ copies/μg total RNA (n = 5), respectively. The expression levels of *CasAQP*-1 at stage Z7-8 and Z8-megalopa were slightly higher with 3.3 ± 1.8 x 10^6^ copies/μg total RNA (n = 6) and 3.6 ± 1.3 x 10^6^ copies/μg total RNA (n = 6). These values did not significantly differ from those measured at stages Z2-3 and Z5-6. Larvae at stages Z2-3, Z3-4, and Z5-6 exposed to ASW at 15 ppt showed significantly higher expression of *CasAQP-1* compared to those exposed to 30 ppt.

#### *AK* expression

**Figure 5:**
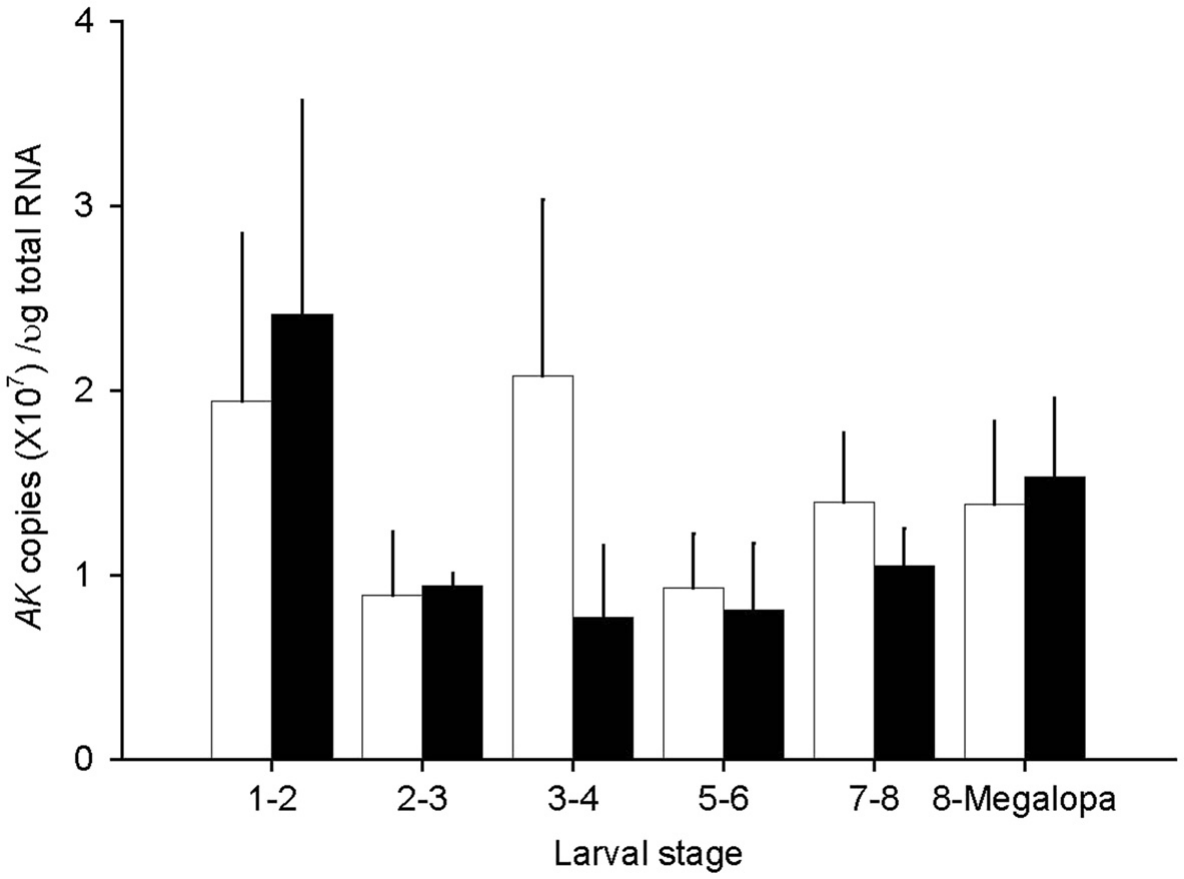
**Quantitative PCR (qRT-PCR) assays of*CasAK*expression in larvae at various developmental stages exposed to the ASW at 30 (open bar) and 15 (closed bar) ppt.** The data are presented as mean ± 1 SE (n = 5–9). No statistical significance was found either between the two treated groups or among the different larval stages in the same treatment.

The expression of *AK* in the larvae exposed to ASW at 30 ppt was relatively consistent throughout the larval stages with a two-fold difference ranging from the lowest level of 0.9 ± 0.3 to the highest level of 1.9 ± 0.9 x 10^6^ copies/μg total RNA (Figure 5). The expression of *AK* in the larvae exposed to ASW at 15 ppt was also similarly consistent throughout the larval stages with a three-fold difference with the lowest of 0.8 ± 0.4 to the highest amounts of 2.4 ± 1.2 x 10^6^ copies/μg total RNA. Indeed, *AK* expression did not differ among the larval stages exposed to ASW at 30 ppt and 15 ppt.

## Discussion

In this study, we isolated the full length cDNA of *AQP-1* from the hypodermis of *C. sapidus* and examined its mRNA expression level in various larval stages in response to hyposalinity.

*CasAQP- 1* cDNA is a 5^′^ TOP mRNA with two TOP sites, one in the 5^′^ UTR and another in the 3′ UTR. These have been shown to be critical for translational control. The 5^′^ TOP found in ribosomal elongation factors is known to be the target of ripampicin, resulting in repression under the stressful, suboptimal growth conditions [17]. Thus, the presence of a TOP site in the 5^′^UTR of *CasAQP-1* implies that upstream signaling pathways and other trans-acting factors regulate its translation [18]. The functional significance of 3^′^TOP is not yet understood.

The ORF of *Callinectes AQP-1* encodes a deduced protein of 238 aa containing two NPA motifs forming the water pore. Four putative phosphorylation sites are predicted in the deduced CasAQP-1, whereas S_228_ is the only site, known to be phophorylated and involved in AQP trafficking [19,20]. The expression pattern of *CasAQP-1* is ubiquitous. In decapod crustaceans, the hepatopancreas and gills are known as the water uptake and osmoregulatory sites, respectively, and these tissues displayed differential expression of *CasAQP-1* with higher expression in the former and lower expression in the latter. Due to the ubiquitous expression of *CasAQP-1* in all tissues examined, and also its small size, we used whole larvae for determining its expression in response to exposure of larvae to different salinities.

The larvae exposed to a constant salinity of 30 ppt exhibited changes in *CasAQP*-*1* expression changes during development, compared to a relatively consistent *AK* expression. Z1-2 and 7–8 larvae have the highest expression of *CasAQP-1*, while Z3-4 and 5–6 larvae showed the lowest expression. Our data imply that *C. sapidus* larvae may undergo endogenous changes in the expression level of *CasAQP-1* during their larval development.

In addition, each of these larval stages shows differential responses when exposed to hyposalinity (15 ppt). Z2-3, Z3-4, and Z5-6 larvae respond by over-expression of *CasAQP-1*, whereas the rest of the larval stages remained at similar levels of expression, compared to the controls exposed to 30 ppt. Because our expression data were obtained after 96 hrs exposure to hyposalinity, we are not certain when the initial response of the *CasAQP-1* upregulation occurs. In contrast to our data, *AQP* expression in the gills of the green crab, *C. maenas*, exposed to 10–15 ppt for 15 days steadily declined, and did not significantly differ from that of the control at 32 ppt [8]. Interestingly, a similar discrepancy was noted in fish in that the expression of *AQP1* of the kidney of animals diverges in response to exposure to hypersalinity and hyposalinity. On the other hand, European eels and black porgy exposed to hypersalinity reduce *AQP-1* expression in the kidney [21,22], whereas Atlantic salmon increase its expression in the kidney [13].

AQPs belonging to a superfamily of MIPs channels passive permeation of water molecules across cellular membranes of bacteria, plants, and animals [23,24]. Thirteen paralogs of AQPs found in mammals show often very specific to a particular cell type, while AQP 1, 3, 7 and 9 are found in various organs including kidney, red blood cells, eyes, ears and lungs [25]. Water permeability is endowed by activation or deactivation of AQPs through phosphorylation or translocation in and out of the cell membrane [26]. Most studies have focused on vertebrate AQPs, particularly euryhaline teleosts’ *AQP 1* as they are capable of maintaining fluid homeostasis against fluctuating salinity conditions. In most fish, two paralogs of *AQP 1* are found: *AQP 1aa* and *1ab* in the kidney, although zebrafish kidney possesses only *AQP 1* aa [27].

Much less is known about invertebrate AQPs, although putative AQP homologues are found in genomic databases. In insects, AQPs include the *Drosophila* integral protein (DRIPs) family with two NPA motifs specific for water transport [28], the *Drosophila* Big Brain gene (DmBiB) family with an extended C-terminal tail similar to human AQP 4, and the PRIP family closely related to DRIPs [29]. Genomic data of *Caenorhabditis elegans* reveal the presence of eight AQPs [30] including three exclusive for water transport, one for glycerol, two for both water and glycerol, and two others not involved in the transport of any solutes examined [31]. Considering the variety of AQPs in other invertebrates, we expect that *C. sapidus* also possesses more than one AQP.

A species-dependent ontogenetic variation in salinity tolerance can drive migratory patterns of dispersal and recruitment of animals. Juvenile and adult crustaceans with high osmoregulatory capacity often occur in estuarine conditions and appear to display two alternative strategies of dispersal and recruitment: retention and export strategies [32]. Those species retaining their larvae in low salinity adult habitats such as *Armases miersii*[33], *Sesarma curacaoense*[34], *Palaemonetes argentines*[35], and *Astacus leptodactylus*[36] show a strong osmoregluatory capacity during the larval stages. On the other hand, *C. sapidus* adopts an export strategy for its larvae with limited osmoreglatory capacity through development of larval stages in coastal shelf or oceanic waters [7]. Consequently, later life stages (megalopae and juveniles) of *C. sapidus* migrate to low salinity areas and colonize estuaries.

The larval development of *C. sapidus* is complex, undergoing 7–8 zoeal stages. *C. sapidus* larvae in this study survived at 15 ppt water for 96 hrs but showed a stunted growth rate at early stages. This indicates that even larvae at earlier zoeal stages do have osmoregulatory capacity, but it is limited. Considering that osmoregulation is an energy-dependent process, it appears that larvae at earlier stages (Z1-4) exposed to 15 ppt compromise growth for survival. However, we are not certain that animals held under different conditions consumed the same amount of food. For this study, we did not directly measure the food intake of each animal, or whether hyposalinity might affect food consumption.

For *C. sapidus* larvae at later stages (Z5 to megalopae), growth increased by stage when larvae were exposed to hyposalinity at 15 ppt. This suggests that larvae at later stages that were exposed to hyposalinity utilized the energy gained from food consumption for growth whereas younger larvae may divert their energy largely for osmoregulation for survival, compromising their growth in the process. This difference in energy diversion could be critical to those larvae at earlier stages with smaller body sizes and hemolymph volumes. Our findings are consistent with reports that *C. sapidus* megalopae collected in the field exhibit a significant osmoregulatory capacity [37]. On the other hand, our data contradict some prior studies which suggest that zoeae have limited osmoregulatory capacity and that metamorphosis results in the immediate appearance of adult-type osmoregulation [2].

In juveniles and adults of decapod crustaceans, gills and guts are the main tissues of iono-and osmoregulation that are mainly regulated by the CHH neuropeptide family [38]. Animals at intermolt stage utilize pleiotrophic CHH and its isoform to increase Na^+^ influx through gill epithelial cells of *Pachygraphsus marmoratus*[39] and hemolymph osmolarity and N^+^ influx in eyestalk-ablated crayfish, *Astacus leptodactylus*[40]. On the other hand, animals during and immediately after ecdysis uptake iso-osmotic water through guts by drinking, the process of which is exerted by the release of gut CHH [41]. The amount of water uptake seems closely associated with the level of CHH concentration in hemolymph, directly resulting in 20-50% molt-related somatic growth over a period of 1–3 hr.

Water uptake occurring during the molting process is recapitulated throughout the life cycle of crustaceans, starting from hatching that coincides with the first molting process. The onset of CHH expression occurs early in the developmental stage and its presence lasts throughout the life cycle [38,42]. CHH mRNA in X-organ cells and neuropeptide in the sinus gland appears at an early eye anlage stage during embryogenesis [43]. As opposed to juveniles and adults, however, the water uptake during hatching is also driven by the release of CHH but not from the endocrine cells located in the fore-and hindguts but those of the abdominal segments of embryos.

The expression pattern of *CasAQP-1* is not determined during embryonic development prior to hatching. *CasAQP-1* expression found in the larvae at Z1-2 implies a possible occurrence of its expression during embryonic development. The activation of vertebrate and mammalian aquaporins by phosphoryation is under the control of various hormones. Thus, it will be interesting to study whether the dramatic water uptake during hatching and molting may be driven by the cascade events of the release of CHH activating crustacean aquaporins.

## Conclusions

We report the ontogenic variation in *CasAQP-1* expression during the larval development of *C. sapidus* at 30 ppt and the induction of its expression at early larval stages in the exposure of hyposalinity. Hyposalinity (15 ppt) compromises only the growth rate during larval development, but not their survival rate. The expression of *CasAQP-1* was common in all tissues obtained from a juvenile crab. It remains to be determined if the increase in *CasAQP-1* expression at later larval stages may have a role in adaptation to hyposalinity.

## Materials and methods

### Animals

*C. sapidus* larvae were collected on the day of hatching and reared in a tank holding 1.5 m^3^ of artificial seawater (ASW) at 30 ppt at 22°C as described previously [44]. During larval rearing, the density of larvae was ~100/L.

### Abrupt exposure of larvae to the ASW at 30 and 15 ppt for 96 hrs

Acute static bioassays were employed to examine salinity tolerance. Two -seven larvae were placed directly into individual wells of a 24-well plate with each well containing 1 ml of filtered ASW at 30 and 15 ppt and kept for 96 hrs at 22-24°C. Larval stages Z1-Z4 were fed 10–15 rotifers/well/day. The larvae at Z5-Z8 were fed 10–15 newly-hatched fresh *Artemia* nauplii daily. The larvae at Z2-3 stages were fed 10–15 one-day old *Artemia* nauplii daily. Z7-8 larvae were fed two days old *Artemia* nauplii daily. The animals were monitored daily for survival and molting for 96 hrs, the time by which the most significant changes in gene expression have been noted in the gills of the green crab (*Carcinus maenas*) exposed to hyposalinity [8]. During the exposure of 96 hrs, some of the larvae underwent molting. Therefore, larval stages were presented as 1–2, 2–3, 3–4, 5–6, 7–8, 8-megalopa in Figures 4 and 5. At the end of exposure, all the larvae retrieved from each well were gently blotted on a tissue paper and placed in a tube. Each larval stage was identified as described [44,45]. Each treatment was hexaplicated and all experiments were replicated using larvae from a total of five independent spawns.

### PCR with degenerate primers

Four degenerate primers were produced (Invitrogen) based on the conserved amino acid sequences of the following aquaporins found in GenBank (Dmel CG9023; AgaP 14229; hsa361_AQP4; bta_281008_AQP4; mmu_11829_AQP4; rno_25293_AQP4; mdo_100010466; gga_421088_AQP4; dre_445293; xla_443817; xla_495037; xtr_448309; dre_335821; dre_559284; hsa_359_AQP2; mcc_711719; cfa486552; bta_539870; xla_378655).

Total RNA was extracted from the tissues using TRIZOl by following the manufacturer’s protocol (Invitrogen) and quantified using a NanoDrop spectrometer (Fisher Scientific). Initially, the total RNA of the hypodermis of *C. sapidus* at premolt was subjected to 5^′^ and 3^′^ RACE cDNA synthesis using the GeneRacer® kit (Invitrogen) following the manufacturer’s protocol. The first amplicon (the expected size, 330 bp) was obtained using a two-step PCR method: 1) touch-down (TD)-PCR and 2) nested PCR, as described [46-48]. In brief, for the TD-PCR, hypodermis cDNA was amplified using Advantage Taq (BD Biosciences) with a combination of primers of AQPdF1 and CasAQPdR1. PCR conditions were as follows: initial denaturation at 94°C for 2.5 min; 94°C, 30 sec, 44°C, 30 sec, 72°C, 1 min, repeated for 3 cycles; 94°C, 30 sec, 44°C, 30 sec, 72°C, 1 min, repeated 3 cycles; 94°C, 30 sec, 42°C, 30 sec., 72°C, 1 min, repeated 3 cycles; 94°C, 30 sec, 40°C, 30 sec, 72°C, 1 min, repeated 3 cycles; 94°C, 30 sec, 45°C, 30 sec, 72°C, 1 min, repeated for 25 cycles and extended 7 min at 72°C. The TD-PCR products were diluted 10 times in sterilized water and then served as the template for a nested PCR with CasAQPdF2 and CasAQPdR2. Eppendorf Taq polymerase was used for the nested PCR at the following conditions: initial denaturation at 94°C for 2.5 min; 94°C, 30 sec, 50°C, 30 sec, 70°C, 1 min, repeated for 35 cycles and extended 7 min at 70°C. The procedures of DNA extraction and subcloning into a pGEM®-T Easy vector (Promega) for sequencing were described previously [46-48].

### 5^′^ and 3^′^ Rapid Amplification of cDNA Ends (RACE) of *C. sapidus* aquaporin

The TD-PCRs were performed with primers CasAQP-1-5R1 and CasAQP-1-3 F1 (Table 1) and the corresponding manufacturer’s primers respectively (Invitrogen), using conditions stated above except for the annealing temperatures: 54^o^, 52^o^, and 50^o^, at a final temperature of 55°C for 1 min extension. The nested PCRs for 5^′^ and 3^′^ RACE were carried out with 10-fold diluted TD-PCR product as template(s) and a combination of primers of CasAQP-1-5R2 and CasAQP-1-3 F2 (Table 1) with 5^′^ nested primer and 3^′^ nested primer of the manufacturer’s primers, respectively. The reaction was amplified at 58°C for annealing and 70°C for 1 min extension. The remaining cloning and sequencing procedures were as described above.

### Spatial expression pattern of *CasAQP-1* in various tissue cDNAs of a juvenile female

Sample cDNAs were prepared from the dissection of a juvenile female at intermolt stage that was raised in 15 ppt salinity. Each tissue cDNA (1.5 μg total RNA) was diluted with sterilized water at the final concentration of 12.5 ng total RNA/ μl. One μl was amplified with CasAQP-1-3F1 and CasAQP-1- 5R1 (listed in Table 1) at 60°C annealing temperature for 35 cycles with Taq polymerase (Eppendorf). The *arginine kinase (AK)* gene was used as a reference gene, as reported elsewhere [47,49]. After electrophoresis on a 1.5% agarose gel containing ethidium bromide, the gel was visualized and digitally-photographed using a Kodak-gel documentation system (Kodak).

### Sequence analyses

The cDNA sequence was analyzed using an ORF finder program (http://www.ncbi.nlm.nih.gov/gorf/gorf.html). The RNA regulatory motifs terminal oligopyrimidine tract (TOP) and upstream ORF (uORF) were predicted using RegRNA [50] (http://regrna.mbc.nctu.edu.tw). The signal peptide of the deduced aa of CasAQP was examined using SignalP 3.0 Server (http://www.cbs.dtu.dk/services/SignalP). The prediction of the phosphorylation sites was performed using NetPhos 2. 0 Server (http://www.cbs.dtu.dk/services/NetPhos/). Potential kinase specific phosphorylation was predicted using NetPhosK 1.0 Server (http://www.cbs.dtu.dk/services/NetPhosK/). The sequence homology was examined using the BLAST network server (blast.ncbi.nih.gov/Blast.cgi). Multiple protein sequences were aligned using ClustalW (http://www.genome.ad.jp).

The deduced aa sequence of CasAQP-1 was formatted into PDB sequences using the Phyre program (http://www.sbg.bio.ic.ac.uk/Phyre², c1mgA (PDB) [51] and viewed using the Jmol program (Version 1.2r3pre) for tertiary structure prediction.

A phylogram was constructed using the neighbor-joining method with 15 deduced amino acid sequences of aquaporin (http://www.phylogeny.fr) [52]. The conserved domain was searched using the conserved domain database (http://www.ncbi.nlm.nih.gov/Structure/cdd) [53].

### Quantitative RT-PCR (qRT-PCR) analysis

The expression of *CasAQP-1* in the samples was determined using a qRT-PCR assay with each sample cDNA containing 25 ng total RNA. Primers for the assays are listed in Table 1. The qRT-PCR standard of *CasAQP-1 and AK* was prepared as described [47-49,54-56]. The level of *AK* expression was examined as a reference gene with an *AK* standard that was generated similarly to that of *CasAQP-1*. The data were presented as mean ± 1SE copies/μg total RNA.

### Statistical analysis

All results represent mean ± 1SE (n), in which n is the number of replicates. GraphPad InStat 3 program (GraphPad Software, Inc) was used to evaluate the statistical significance of the data. Statistical significance among the different larval stages at the exposure to 30 or 15 ppt was determined using one-way ANOVA with post-hoc Tukey-Kramer multiple comparison tests and was accepted at *P* <0.05. The difference of the same larval stage at two different salinities was calculated using Student’s *t* test.

## Competing interests

Authors declare no conflicting interests.

## Authors’ contributions

JSC for cloning *AQP-1*, qRT-PCR assays and data analyses; JSC, JSP, and MBO for larval experimental design; MB for salinity exposure study; LM for salinity exposure study, RNA extraction and cDNA synthesis; JSC & JSP for funding; JSC, JSP & MBO for writing the manuscript. All authors read and approved the final manuscript.
